# Congenital Sternal Cleft along with Persistent Left-Sided Superior Vena Cava: A Rare Presentation

**DOI:** 10.1155/2013/192478

**Published:** 2013-06-13

**Authors:** Anindya Kumar Saha, Syamal Kumar Sardar, Amitava Sur

**Affiliations:** ^1^RMO Cum Clinical Tutor, Department of Neonatology, IPGME&R-SSKM Hospital, A. J. C. Bose Road, Kolkata 700020, India; ^2^Department of Neonatology, IPGME&R-SSKM Hospital, A. J. C. Bose Road, Kolkata, PIN-700020, India

## Abstract

Congenital sternal cleft is a rare abnormality resulting from fusion failure of sternum. It occurs in isolation or along with defects of abdominal wall, diaphragm, pericardium, and heart. Early surgical correction is required to protect the underlying structures for risk of cardiac compression. Here we report a case of 20-day female child presenting with congenital sternal cleft associated with multiple congenital heart disease and left-sided superior vena cava. She was operated by the cardiothoracic surgical team successfully and is doing well on followup. We discuss this rare case, imaging studies, and surgical strategy.

## 1. Case History

**Figure 1:**
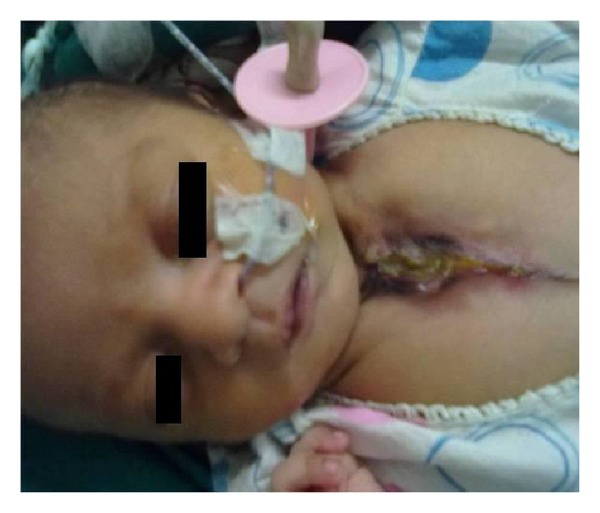
Preoperative appearance of the baby depression over chest wall.

**Figure 2:**
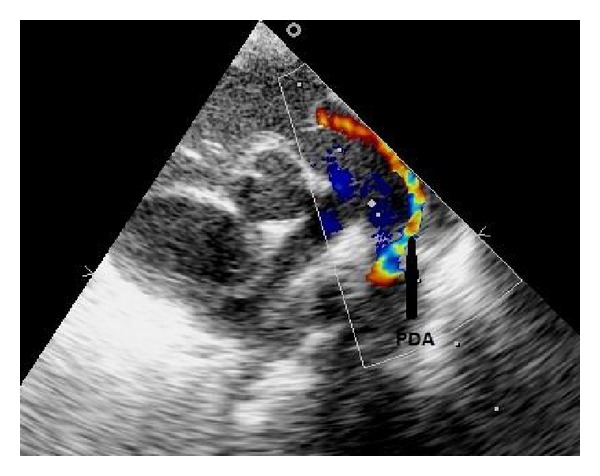
Transthoracic echocardiogram, parasternal short axis view showing patent ductus arteriosus.

A 20-day old female baby, with a birth weight of 2.2 kg, born by caesarean section at term to primi mother was referred to our neonatal intensive care unit with complain of a central depression over the chest wall in inspiration with movements with respiration. There was a prominent midline raphe over the skin starting from anterior neck and visible cardiac pulsations in the suprasternal region (Figure 1). Examination revealed a central bony defect under the depression along the midline and a grade II systolic murmur over the precordium. The baby was hemodynamically stable. Chest radiograph revealed an enlarged thymic shadow. Cranial and abdominal ultrasounds were normal. Transthoracic echocardiography revealed a 3 mm ostium secondum atrial septal defect (ASD) with left-to-right shunt and a 2 mm patent ductus arteriosus (PDA) (Figure 2). The child also had a left-sided superior vena cava (SVC) draining to a dilated coronary sinus with absent right SVC. The patient underwent primary surgical correction at 25 days of life.

**Figure 3:**
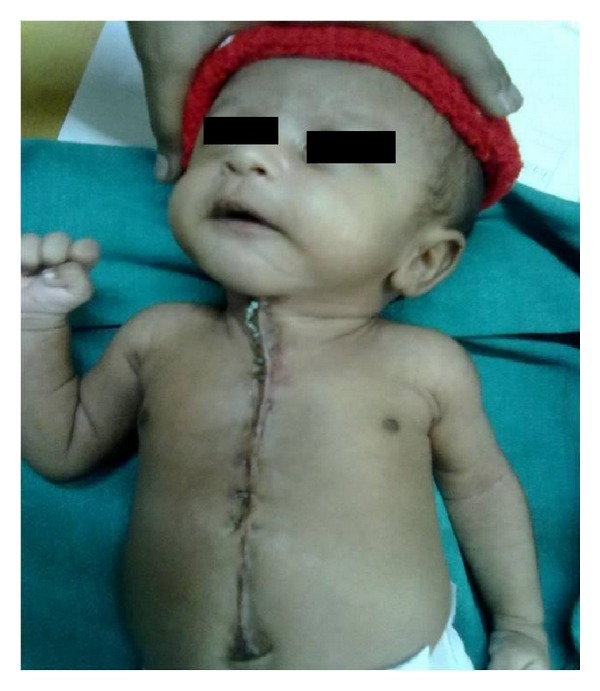
Postoperative skin.

She was operated in supine position through a midline chest incision and the sternal cleft was found. A thickened fibrous raphe was seen tethering the manubrium to the intact pericardial sac. The raphe was excised thus also releasing the neck of the child. The right lobe of thymus was resected. Absent right SVC was confirmed. A U-shaped cartilaginous bar, 3.7 cm long, was found joining the margins of the sternum from middle to lower part. This bar was excised along its entire length, and the two separated parts of the sternum were pulled across and approximated gradually by four ethicon sutures over the underlying mediastinal structures. This position was maintained for 5 minutes to look for any hemodynamic compromise. As there was no instability, the sutures were tied and the wound closed in two layers. The baby was mechanically ventilated in the immediate postoperative period and weaned to room air after 24 hours. There were no episodes of hemodynamic compromise and inotropes were not required. She was started on feeds on the second postoperative day and reached full feeds within 72 hours. She was discharged home and was doing well in the first-week followup (Figure 3). Her parents have been advised on scheduled followup at the neonatal and cardiothoracic department.

## 2. Discussion

Congenital sternal cleft is a rare anomaly which might occur in isolation or along with defects of abdominal wall, diaphragm, pericardium, and heart, and early diagnosis and surgical correction gives the infant the best chance of survival [1, 2]. Its incidence is reported to be fewer than 1 in 100000 live births whereas persistent left SVC is the most common congenital thoracic venous anomaly with a prevalence of 3–5 in 1000 in general population [3, 4]. The combination of these two entities with other heart diseases obviously makes it extreme rare. In embryonic life, the sternum originates from the lateral plate mesoderm. Cells from two bands of mesoderm on either side of the anterior chest wall migrate toward the midline and become fused by the tenth week, thus forming the sternum. The manubrium is formed by primordia between the ventral ends of the developing clavicles. The process of fusion of sternal bars proceeds in a cephalocaudal direction. Very rarely, the sternal bars fail to join in the midline, which results in a complete sternal cleft [5]. The aetiology of cleft sternum remains obscure. Sternal cleft can be classified into three major groups: (a) cleft sternum without associated anomalies, (b) thoracic or true ectopia cordis with various degrees of cleft sternum with the heart lying outside the chest wall, and (c) thoracoabdominal ectopia cordis referred to as Cantrell pentalogy [1]. Cleft sternum without associated anomaly or isolated sternal cleft ranges from partial to complete. Our patient had a partial sternal cleft which presents with skin coverage of midline defect with an intact pericardium and normal diaphragm. This patient also had other anomalies like ASD, PDA, and persistent left SVC which makes it rare, even rarer than pentalogy of Cantrell whose incidence is 1 in 100,000 of life births [6, 7]. In this challenging anomaly, the underlying midiastinal structures (most especially the heart and great vessels) may easily be injured by external trauma. In addition, the deformity is cosmetically unpleasant and quite alarming to the young patient and his or her family. The nature of surgical reconstruction also varies with the age of presentation, and the methods involve primary apposition or reconstruction with bone grafts, pectoralis grafts, and chondrotome. The target is to provide adequate protection of thoracic organs, to maintain growth of chest wall and to avoid the use of any prosthetic material whenever possible. In neonates primary closure is attempted due to the cartilaginous nature of sternum and flexibility of chest wall.

Presence of persistent left SVC (PLSVC) diagnosed incidentally has made the presentation unique. It occurs due to failure of normal regression of the left superior cardinal vein [8]. The most common subtype results in the presence of both right and left SVC. More rarely caudal right superior vein regresses leading to absence of right SVC with PLSVC. In 80–90% cases PLSVC drains into right atrium via coronary sinus and is of no hemodynamic significance which is seen in this case. Almost 40% of patients with PLSVC can have variety of cardiac anomalies like ASD, bicuspid aortic valve, and so forth, more commonly seen with absent right SVC [9]. This index case had such absent right SVC with ASD and PDA. PLSVC may be associated with anatomical and architectural defect of sinus node for which the baby needs long-term followup. However PLSVC has got various practical implications in situations like access to right side of the heart or pulmonary vasculature via left subclavian vein or pace maker implantation. It is also a relative contraindication to the administration of retrograde cardioplegia [8].

 Here we have described a rare presentation of isolated sternal cleft with persistent left SVC, ASD, and PDA with good cardiac function and rhythm, where primary closure was successfully done at the age of 25 days without any cardiovascular compromise at the postoperative period. We understand that the child needs long-time followup regarding cardiac and pulmonary function. But with the uneventful postoperative period the future of this patient looks promising.
